# Microplastics in Human Ovarian Follicular Fluid and Female Fertility

**DOI:** 10.7759/cureus.116365

**Published:** 2026-09-16

**Authors:** Iwona Kaliciak

**Affiliations:** 1 Department of Medicine, Clinical University Hospital Poznań, Poznań, POL

**Keywords:** assisted reproduction, female fertility, follicular fluid, microplastics, nanoplastics, oocyte quality, ovarian reserve, reproductive toxicology

## Abstract

Microplastics and nanoplastics are increasingly reported in human biological matrices, raising concern about the exposure of the ovarian follicle during oocyte maturation. This narrative review evaluated evidence on plastic particles or polymer mass in human ovarian follicular fluid and their potential relevance to female fertility and assisted reproductive technology (ART). PubMed, including the Medical Literature Analysis and Retrieval System Online (MEDLINE); publisher records; reference lists; and citing articles were searched through July 28, 2026. Six full human observational studies and three preliminary reports were identified, demonstrating the limited size of the current evidence base. The full studies detected plastic-derived particles or polymer mass in human follicular fluid, although reported concentrations and polymer profiles differed substantially across analytical platforms. Several small studies associated higher polyethylene (PE) or polyvinyl chloride (PVC) burdens with lower fertilization rates, higher follicle-stimulating hormone (FSH) levels, or diminished ovarian reserve (DOR), while the largest case-control study reported the strongest association for polyamide 66 (PA66). These findings have not been independently replicated and remain observational. Cross-sectional designs, small and selected infertility-clinic populations, residual confounding, possible procedural contamination, nonequivalent measurement units, and the absence of standardized analytical methods preclude causal interpretation and quantitative comparison across studies. Animal and cell studies provide mechanistic plausibility but frequently use exposure conditions that may not represent human follicular exposure. Current evidence therefore supports the presence of plastic-derived material in the human follicular microenvironment but does not establish that microplastics cause infertility or that follicular-fluid testing has clinical utility. Prospective multicenter studies using standardized contamination controls, harmonized exposure measurements, and preregistered reproductive outcomes are required.

## Introduction and background

Ovarian follicular fluid is the immediate extracellular environment of the cumulus-oocyte complex. It comprises plasma-derived constituents and secretions from theca and granulosa cells that support communication with the oocyte. Its composition reflects systemic inputs and local follicular physiology and can influence the nuclear and cytoplasmic maturation of the oocyte [1]. Because follicular fluid is collected during oocyte retrieval, assisted reproductive technology (ART) provides a rare opportunity to examine an otherwise inaccessible human reproductive compartment [1].

Microplastics are commonly defined as plastic particles smaller than 5 mm, whereas nanoplastics usually refer to particles below 1 µm, although size boundaries and terminology remain inconsistent [2]. Human exposure is plausible through ingestion, inhalation, and dermal contact. Plastic-derived particles or polymer mass have been reported in blood and multiple tissues, but detection alone does not establish toxicological significance [3,4]. Entry into an ovarian follicle is biologically plausible because the blood-follicle barrier is size- and charge-selective rather than absolute [5]. Translocation may also depend on protein coronas, surface weathering, particle shape, and inflammatory or vascular conditions. These considerations are particularly relevant to small particles, yet current human analytical methods detect different and only partially overlapping size fractions [2-5].

The first reports of microplastics in human follicular fluid appeared only recently [6,7]. The evidence base has since expanded from a reviewed preprint and meeting abstracts to peer-reviewed observational studies evaluating fertilization and diminished ovarian reserve (DOR) [8-14]. Interpretation has nevertheless been complicated by small samples, heterogeneous infertility diagnoses, dissimilar analytical platforms, and the frequent blending of human associations with experimental causation [6-14]. The aims of this review are to synthesize direct human follicular-fluid evidence through July 28, 2026; appraise analytical validity and contamination control; summarize relevant animal and in vitro mechanisms; define what can and cannot currently be inferred for female fertility and ART; and develop a platform-aware exposure model and staged framework for translation from detection to clinical utility.

## Review

Review methodology

Search Strategy and Keywords

PubMed, including the Medical Literature Analysis and Retrieval System Online (MEDLINE), was searched from database inception through July 28, 2026. The principal keyword blocks were “microplastic,” “microplastics,” “nanoplastic,” “nanoplastics,” “plastic particle,” “polyethylene,” “polyvinyl chloride,” “polyamide,” “polystyrene,” “polypropylene,” “follicular fluid,” “ovary,” “ovarian,” “oocyte,” “granulosa,” “cumulus,” “fertility,” “in vitro fertilization,” “assisted reproduction,” and “diminished ovarian reserve.” Terms describing plastic particles or polymers were combined with terms describing the ovarian compartment or reproductive outcomes. A focused human search combined (“microplastic” OR “nanoplastic” OR “plastic particle”) with (“follicular fluid” OR “cumulus” OR “granulosa”) and (“fertility” OR “oocyte” OR “in vitro fertilization” OR “ovarian reserve”). Candidate records were checked against publisher pages, digital object identifier (DOI) metadata, reference lists, and citing articles. Citation-chain searching was used to identify relevant records in journals indexed by Scopus or Web of Science. Recent reviews of human microplastic analysis and female reproductive toxicology were used to identify additional primary studies.

Inclusion and Exclusion Criteria

Human records were eligible for the direct-evidence synthesis when they reported primary measurements of plastic particles, nanoplastic-range material, or polymer-specific mass in human ovarian follicular fluid. Full peer-reviewed articles, reviewed preprints, and meeting abstracts with interpretable methods and results were eligible; full articles formed the principal synthesis, whereas preliminary records were explicitly labeled and given lower evidentiary weight. Experimental studies were eligible when they examined ovarian tissue, granulosa or cumulus cells, follicles, oocytes, fertilization, or embryo development in mammalian models. English-language reports available as full text or sufficiently detailed abstracts were considered.

Records were excluded from the direct-evidence synthesis if they were reviews, editorials, commentaries, duplicate reports, non-mammalian studies, studies without an ovarian or reproductive endpoint, studies of plastic-associated chemicals without particle or polymer measurement, or reports that did not provide sufficient methodological detail to determine the analyzed matrix or exposure measure. Reviews and studies of non-particle endocrine-disrupting chemicals could nevertheless be retained as contextual sources when they informed analytical interpretation, mixture effects, or clinical outcome assessment.

Study Selection and Evidence Synthesis

Records identified through the database search, publisher records, reference lists, and citation-chain searching were consolidated before eligibility assessment. Potential duplicates were identified by comparing titles, digital object identifiers, author lists, publication years, study populations, and reported sample sizes. Duplicate bibliographic records were removed. When a preliminary report and a subsequent full article described the same or an overlapping study population, the full article was used as the principal source, while the preliminary report was retained only if it contained relevant information not available in the full publication or if no full article had been published by the review cutoff date.

Records were screened sequentially by title and abstract and then by full text when they appeared potentially eligible. Screening, eligibility assessment, and data extraction were not performed independently in duplicate. Full-text records were excluded from the direct human-evidence synthesis when they did not measure plastic particles or polymer-specific mass in human ovarian follicular fluid, did not report an eligible ovarian or reproductive endpoint, examined only plastic-associated chemicals without measuring particles or polymers, represented a review or commentary rather than primary research, duplicated or substantially overlapped a more complete report, or provided insufficient methodological information to determine the analyzed matrix or exposure measure. Experimental studies were excluded from the mechanistic synthesis when they used non-mammalian models or did not evaluate an ovarian, follicular, granulosa-cell, cumulus-cell, oocyte, fertilization, or embryo-development endpoint.

Six full human observational studies and three preliminary records met the direct human-evidence criteria. A formal record-level screening log was not maintained; therefore, reliable counts of records retrieved, excluded during title and abstract screening, and excluded after full-text assessment could not be reconstructed retrospectively. The absence of a PRISMA-style screening flow is acknowledged as a limitation of the narrative-review methodology.

For each included record, the review extracted study design, population, sample size, biological matrix, analytical platform, detectable size window, contamination-control procedures, polymer profile, exposure unit, reproductive endpoint, statistical approach, and principal interpretive limitations. Human observational evidence was synthesized separately from animal and in vitro evidence. Qualitative evidentiary weight was based on publication status, sample size, study design, contamination control, the molecular confirmation of polymer identity, adjustment for confounding, temporal ordering, and clinical relevance.

This review was designed as a narrative rather than a systematic review. No prospective protocol was prepared or registered, independent duplicate screening and extraction were not undertaken, and no formal risk-of-bias instrument was applied. No meta-analysis was attempted because the exposure metrics, particle-size windows, polymer definitions, analytical platforms, and reproductive outcomes were not sufficiently comparable.

Human evidence

Emergence of Direct Follicular-Fluid Evidence

The earliest direct report was a 2023 eLife Reviewed Preprint by Grechi et al., which identified microplastics in follicular fluid from seven women and complemented these observations with bovine follicular-fluid and oocyte experiments [6]. The study was important as a proof of concept but remains a reviewed preprint rather than a journal version of record. A 2024 Human Reproduction conference abstract subsequently reported pyrolysis-gas chromatography/mass spectrometry (Py-GC/MS) findings in cumulus granulosa-cell samples from 16 patients and follicular fluid from two patients [7]. Polymer mass was higher in cumulus-cell preparations than in the two fluid samples, and the abstract reported an association with cleavage rather than fertilization or good-quality embryo rate. Its very small follicular-fluid sample and abstract-only methods preclude firm inference [6,7].

In 2025, Montano et al. published a full peer-reviewed clinical study specifically evaluating human follicular fluid [8]. Eighteen women undergoing ART in Italy contributed blood-free fluid from the first aspirated follicle. Scanning electron microscopy (SEM) with energy-dispersive X-ray analysis (EDX), collectively referred to as SEM-EDX, identified putative microplastics smaller than 10 µm in 14 of 18 samples, with an average of 2,191 particles/mL, a range from zero to 7,181 particles/mL, and a mean diameter of 4.48 µm. Particle concentration correlated positively with follicle-stimulating hormone (FSH) but not with anti-Müllerian hormone (AMH), fertilization, miscarriage, or live birth. The study used strong collection and blank procedures, but SEM-EDX did not provide polymer-specific molecular identification. The FSH association was unadjusted within a small sample and should be viewed as hypothesis-generating [8].

Ni et al. evaluated follicular fluid from 19 women using laser direct infrared (LDIR) imaging and Py-GC/MS [9]. LDIR classified 1,739 of 7,956 analyzed particles as microplastics and described 30 polymer types; the dominant assignments included chlorinated polyethylene (PE), fluorosilicone rubber, polyvinyl chloride (PVC), butadiene rubber, and styrene-butadiene-styrene. Py-GC/MS of five randomly selected samples detected polyethylene (PE), PVC, polypropylene (PP), and polystyrene (PS), with PE contributing the largest mass. The human component was principally descriptive. Separate in vitro mouse-oocyte experiments suggested that multiple fluorescent plastic particle types could impair maturation, but those experiments cannot establish that the complex human follicular-fluid mixture caused an effect [9].

Wang et al. used Raman microspectroscopy in 44 women receiving ART [10]. PE was the most frequently detected polymer, with a reported detection rate of 86.4%. Higher follicular-fluid PE counts were inversely correlated with fertilization rate (reported correlation coefficient, -0.407; p = 0.007), and metabolomic analyses highlighted pathways related to ferroptosis and steroidogenesis. In mice, PE exposure increased ovarian oxidative and inflammatory signals and reduced oocyte yield and fertilization. The human result remains observational: an exposure measured at oocyte retrieval may correlate with an unmeasured characteristic of the patient, treatment, laboratory, or follicle, while the mouse experiment tests a selected polymer under controlled exposure rather than reproducing the human exposure mixture [10].

A 2025 meeting abstract by Gomez-Sanchez et al. reported an LDIR detection of several polymers in follicular fluid from 25 women and seminal fluid from 18 men [11]. Polyamide, polyurethane, and PE were each reported in more than half of follicular-fluid samples; other polymers included polytetrafluoroethylene, polyethylene terephthalate (PET), PP, PVC, and polylactic acid. The investigators used glass storage, potassium hydroxide digestion, and container controls. No clinical outcome analysis was presented, and the report should be regarded as preliminary until full methods and blank data are available [11].

The Couple-Based In Vitro Fertilization (IVF) Study

Kong et al. examined follicular fluid and seminal plasma from 51 couples undergoing conventional in vitro fertilization (IVF) [12]. Eligibility limited the female partner to tubal-factor infertility and the male partner to normal semen parameters; women with DOR and couples with low fertilization were excluded. This restriction not only reduced some clinical heterogeneity but also narrowed generalizability and may have attenuated adverse-outcome associations. Py-GC/MS detected PE and PVC in every follicular-fluid sample, with mean concentrations of 1.21 and 1.85 µg/g, respectively. Higher tertiles of either polymer were associated with lower normal fertilization rates. Follicular-fluid burdens were not consistently associated with ovarian-reserve indices, implantation, or clinical pregnancy. PVC in seminal plasma was inversely associated with sperm motility [12].

The analytical workflow removed fragments larger than 1 µm by stainless steel filtration before Py-GC/MS, and the authors described the retained signal as nanoplastic-range polymer mass. Procedural blanks for follicular fluid and seminal plasma were negative, recovery was reported within 80%-120%, plastic consumables were profiled, and glassware underwent high-temperature preparation. These measures strengthen confidence that PE and PVC signals were not obvious laboratory artifacts. However, Py-GC/MS measures polymer-specific mass after thermal decomposition; it does not count intact particles, identify morphology, or prove that all measured mass consisted of discrete particles below 1 µm. The term “nanoplastics” therefore depends on the sample-preparation logic and cannot be interpreted as direct particle-size measurement [12].

Diminished Ovarian Reserve Studies in 2026

Zhou et al. conducted a case-control study of 25 women with DOR and 20 women with tubal-factor infertility [13]. Py-GC/MS, LDIR, and SEM provided complementary mass, chemical-imaging, and morphological information. Microplastics were found in every follicular-fluid sample. Total polymer mass was higher in the DOR group than in controls (30.63 versus 18.48 µg/g), with higher PE, PP, and PVC concentrations. In adjusted models, PE, PVC, and total microplastic concentrations were associated with DOR; total, PE, and PVC burdens were inversely related to AMH, and total burden was inversely related to antral follicle count (AFC). The same publication included mouse exposure experiments supporting ovarian toxicity [13].

This study is the first full report to describe an association between follicular-fluid polymer burden and a clinically defined ovarian-reserve phenotype. Nonetheless, DOR status and follicular exposure were measured in the same treatment cycle. Reduced follicle number or altered follicular physiology could influence local concentration or clearance, and residual confounding by age, diet, occupation, medication, plastic use, or infertility history remains possible. Odds ratios from a small case-control sample do not establish incidence or temporality [13].

Si et al. subsequently analyzed follicular-fluid microplastics and nanoplastics in 110 women with DOR and 110 age-matched controls, the largest human study available by the review cutoff [14]. Multiple polymers were detected. Polyamide 66 (PA66) showed the strongest reported association with DOR, while PS and PVC concentrations were also higher in cases. Experimental components linked mixed-particle exposure to ovarian-reserve impairment, intestinal-barrier disruption, gut-microbiome changes, reduced *Parabacteroides goldsteinii* (*P. goldsteinii*) and 7-ketolithocholic acid, and the activation of the phosphoinositide 3-kinase (PI3K), protein kinase B (AKT), and mechanistic target of rapamycin (mTOR) signaling pathway in granulosa cells. Microbial supplementation ameliorated several exposure-associated phenotypes in mice [14].

The larger sample and age matching improve precision relative to earlier cohorts, but the human evidence remains case-control and cross-sectional. The microbiome intervention demonstrates reversibility in the specific mouse model, not in women, and should not be interpreted as a validated fertility treatment. Independent replication, detailed exposure-source assessment, and prospective sampling before ovarian decline are needed [14].

Human Cumulus-Cell Imaging

Bozzer et al. used cadmium selenide quantum dot-labeled PE particles with a mean diameter of approximately 233 nm to investigate internalization by human cumulus cells [15]. Spectral confocal microscopy and synchrotron X-ray fluorescence provided orthogonal localization of the engineered tracer. The study demonstrates that human cumulus cells can internalize a defined PE nanoplastic model under experimental conditions. It does not show environmental PE within patient-derived cells, quantify real-world follicular exposure, or establish functional toxicity [15].

Analytical methods and contamination control

What the Major Platforms Measure

The apparent abundance and polymer profile in follicular fluid depend strongly on the analytical platform. Scanning electron microscopy (SEM) visualizes surface morphology at high spatial resolution, and energy-dispersive X-ray analysis (EDX) characterizes elemental composition. This combination can distinguish some organic-appearing particles from mineral material, but EDX provides elemental rather than molecular information. Because elemental composition is not polymer-specific, polymer assignment remains uncertain without vibrational or thermal confirmation [16-18].

Raman microspectroscopy and Fourier-transform infrared approaches, including LDIR, compare vibrational spectra with reference libraries. They can identify polymer chemistry and provide particle counts, dimensions, and morphology within instrument-specific detection windows. Raman can analyze smaller particles than conventional infrared imaging but is vulnerable to fluorescence and lengthy acquisition. Infrared imaging can process larger areas rapidly but commonly misses submicron particles and may misclassify degraded, pigmented, or protein-coated particles. Match thresholds and library composition affect reported polymer diversity [16-18].

Py-GC/MS thermally decomposes a sample and quantifies polymer-specific marker compounds. It is sensitive and useful for mass-based measurement in complex biological matrices, but it destroys the particles. Consequently, it cannot directly report number, shape, or size distribution, and closely related polymers or background chemicals may produce overlapping pyrolysis products. Particle-count data and polymer-mass data answer different questions and cannot be directly converted without assumptions about density, shape, and size distribution [16-18].

The studies reviewed here therefore should not be expected to identify identical polymer rankings. A high number of small particles can contribute little mass, whereas a few larger particles can dominate mass. Detection windows are also consequential: filtering out material above 1 µm before thermal analysis enriches a nominal nanoplastic fraction but does not itself measure each retained object. Conversely, an imaging platform with a lower size limit of several micrometers cannot evaluate the submicron fraction, which may have greater potential for cellular uptake than larger particles [4,12,16-18].

Contamination Pathways

Follicular-fluid studies face contamination risks at patient preparation, aspiration, sample storage, digestion, filtration, transfer, and instrumental analysis. Potential sources include collection tubing, syringes, aspiration needles and seals, laboratory air, clothing fibers, gloves, plasticware, membrane filters, water, reagents, and the thermal or spectroscopic system itself. ART laboratories also use multiple disposable products before and after oocyte retrieval. Contamination can create false positives, but overly aggressive digestion or filtration can destroy or remove target polymers and create false negatives [16-18].

Blank controls are therefore essential. Blank samples should be processed in parallel with study samples across collection, preparation, and analysis so that sample-derived particles can be distinguished from contamination. Blank results should be reported explicitly and considered when interpreting or correcting sample values [16]. Recovery testing in matched biological matrices is also important for characterizing losses introduced by digestion and filtration [17,18].

The strongest current human studies used glass or metal contact materials where feasible, covered vessels, low-shedding clothing, clean-air handling, precombusted glassware, recovery testing, and procedural blanks [8,12]. Even these precautions cannot reconstruct contamination that occurs inside commercial clinical consumables before the sample reaches the research workflow. Profiling tubing and containers, collecting rinsate controls, and sampling the first and later follicles separately would help identify procedural contributions [8,12,16].

Reporting and Harmonization

Future studies should report patient-level raw concentrations, blank-corrected and uncorrected values, limits of detection and quantification, spectral match thresholds, particle-size limits, digestion efficiency, recovery, polymer-specific results, and sample volumes. Units should be standardized within method class, such as particles/mL for imaging and µg/g for thermal mass, without implying equivalence. Representative spectra and images should be deposited when privacy and journal policies permit. Orthogonal confirmation is particularly important for uncommon polymers or high prevalence. On the basis of the complementary strengths described in methodological reviews, we propose imaging spectroscopy for particle count, size, shape, and chemical identity, combined with Py-GC/MS for polymer mass and SEM for morphology. Interlaboratory reference materials for biological matrices remain a major unmet need [17,18].

Mechanistic evidence from animal and in vitro studies

Granulosa-Cell Injury, Inflammation, and Cell Death

Granulosa cells maintain the metabolic and endocrine environment required for follicle growth and oocyte maturation [1]. Experimental PS nanoplastics increased reactive oxygen species, apoptosis, and Hippo-pathway disturbance in a human ovarian granulosa-cell line and produced ovarian injury and reduced fertility in exposed mice [19]. In rats, PS microplastics activated nucleotide-binding oligomerization domain-like receptor family pyrin domain-containing protein 3 (NLRP3)/caspase-1-associated pyroptosis and apoptosis in ovarian granulosa cells [20]. Other rodent studies described altered ovarian cytoskeletal proteins, follicular development, hormones, and fertility after PS exposure [21,22]. Co-exposure to PS particles and the plasticizer di(2-ethylhexyl) phthalate increased oxidative stress, deoxyribonucleic acid (DNA) damage, cell-cycle arrest, and necroptosis in mouse granulosa cells [23]. These findings converge on cellular stress and loss of follicular support, but they do not establish which pathways occur at measured human follicular-fluid concentrations [19-23].

Oxidative Stress, Steroidogenesis, and Mitochondrial Function

Oxidative stress is the most recurrent experimental signal. Mouse studies have reported lipid peroxidation, reduced antioxidant capacity, follicular atresia, hormonal changes, and reproductive effects in exposed female mice and offspring [24]. PVC microplastic exposure has additionally been linked to ovarian and intestinal changes and altered gut microbiota in mice [25]. Cultured mouse antral follicles exposed to nanoscale PS or PET showed polymer- and concentration-dependent effects on growth, steroidogenesis, and gene expression [26]. Whole-cycle and follicle-culture experiments also suggest that PS particles can perturb folliculogenesis and endocrine function [27]. The diversity of particles and protocols makes a single threshold impossible to derive [24-27].

Particle exposure may impair oocyte competence through mitochondrial dysfunction.

Oocytes require precisely timed adenosine triphosphate (ATP) production, redox regulation, spindle assembly, and organelle redistribution [1]. In bovine cumulus-oocyte complexes, 50-nm and 200-nm PS particles entered cumulus cells, while the smaller particles showed greater access to the oocyte; exposure delayed maturation and early embryo development [28]. PE microplastics of 10-30 µm impaired meiotic progression and altered mitochondrial distribution and glutathione in a bovine oocyte model [29]. These large particles may act through cumulus contact or extracellular stress rather than direct entry into the oocyte. Engineered PS nanoplastics have also been reported to disrupt granulosa-cell cytoskeletal organization and epigenetic programs during ovarian development [1,28-30].

Environment-Gut-Ovary Signaling

The 2026 DOR study by Si et al. broadened the mechanistic model beyond direct ovarian deposition [14]. In mice, mixed microplastic and nanoplastic exposure was associated with intestinal-barrier damage, gut dysbiosis, the loss of *P. goldsteinii* and its metabolite 7-ketolithocholic acid, and ovarian-reserve impairment. Microbial supplementation ameliorated several phenotypes, while granulosa-cell transcriptomics implicated PI3K/AKT/mTOR signaling. This environment-gut-ovary axis is biologically interesting because systemic inflammation and microbial metabolites can affect ovarian function without requiring every particle to reach the follicle. However, the selected exposure mixture, mouse microbiome, and intervention conditions may not generalize to heterogeneous human exposures [14].

Limits of Mechanistic Extrapolation

Experimental studies often use pristine spherical PS, fluorescent beads, or engineered particles at concentrations chosen to reveal a biological response. Real human exposure includes weathered fragments and fibers, multiple polymers, additives, adsorbed contaminants, biological coronas, and chronic low-dose mixtures. Nominal media concentrations may differ from the dose reaching cells because particles aggregate, sediment, or adhere to vessels. Fluorescent labeling may leach, and mass doses obscure particle-number differences across sizes. Species differences in folliculogenesis, exposure kinetics, ovarian reserve, and ART stimulation further limit extrapolation [4,16-30].

Mechanistic evidence therefore increases plausibility but cannot convert a human cross-sectional association into causation. Its most appropriate use is to identify candidate biomarkers and pre-specify pathways for human studies, such as oxidative damage, steroidogenic disruption, mitochondrial function, inflammatory signaling, or follicular metabolomics [8-14,19-30].

Implications for female fertility and assisted reproductive technology

Ovarian Reserve

AMH, antral follicle count, and basal FSH are markers of oocyte quantity or expected response to stimulation, not direct tests of oocyte quality or natural fecundity [31]. DOR also has variable definitions across clinical practice, despite consensus criteria for poor ovarian response [32]. The positive FSH correlation reported in 18 women [8] and the case-control associations reported in 2026 [13,14] are therefore signals of possible relevance, not proof that particles depleted the primordial follicle pool. Longitudinal studies would need to demonstrate that exposure precedes accelerated AMH or antral-follicle decline, reduced oocyte yield, or earlier menopause [8,13,14,31,32].

Oocyte Competence and Fertilization

Two full human studies associated higher follicular PE, or PE and PVC, with lower fertilization [10,12]. This consistency is notable because fertilization is temporally close to follicular-fluid sampling and has complementary experimental support. Yet, fertilization is influenced by sperm quality, insemination method, oocyte maturity, stimulation, embryology procedures, and laboratory performance. Separately, cumulative combinations of IVF disposables have shown toxicity in a human sperm-motility assay, although that study did not evaluate clinical fertilization [33]. One study excluded couples with low fertilization and used conventional IVF [12], while another studied a broader infertile population [10]. Confirmation requires follicle-level linkage among exposure, the retrieved oocyte, maturity, insemination, and embryo trajectory, with appropriate multilevel models for oocytes nested within women and cycles [10,12,33].

Human follicular fluid already contains mixtures of endocrine-disrupting chemicals associated with impaired oocyte development in experimental assays [34]. Plastic particles may act independently, carry additives or environmental contaminants, or simply mark a broader exposure mixture. Studies that measure particles without common plasticizers, persistent organic pollutants, metals, diet, and occupation risk attributing mixture effects to the polymer signal alone [4,34].

Embryo Development, Pregnancy, and Live Birth

Available studies are underpowered for implantation, miscarriage, clinical pregnancy, and live birth. Kong et al. did not identify consistent implantation or clinical-pregnancy associations [12], and Montano et al. reported no miscarriage or live-birth association in only 18 women [8]. These null findings should not be interpreted as evidence of either safety or harm because few events were available, embryo transfer introduces selection, and treatment decisions may mediate outcomes. Live birth is a clinically important endpoint, but obtaining sufficient power will require multicenter cohorts and careful accounting for cumulative transfers and embryo cryopreservation [8,12].

Clinical Practice

Based on the reviewed direct human evidence, routine follicular-fluid microplastic testing is not currently justified. None of the direct human studies established a validated clinical reference range, an actionable threshold, or an ability of testing to improve treatment decisions [8-15]. Results from different analytical platforms are not interchangeable, and individual counseling could create anxiety in the absence of an evidence-based intervention [16-18]. Although the reduction of unnecessary plastic exposure may be considered for environmental or occupational reasons, current studies do not show that a specific avoidance strategy improves ovarian reserve or ART outcomes [4,8-14]. Likewise, the mouse probiotic intervention reported in 2026 should not be offered as a fertility treatment on current evidence [14].

ART centers can nevertheless support research quality by auditing consumables, documenting aspiration materials and lot numbers, storing research aliquots in validated glass containers, and participating in standardized multicenter protocols; cumulative exposure to IVF disposables is itself an active area of laboratory-safety research [33]. Such measures should be framed as contamination control and exposure science, not as proven clinical therapy [16-18,33].

Integrative interpretation and novel insights

A Measurement-Defined Exposure Construct

Across the current human literature, “follicular-fluid microplastic burden” does not represent a single interchangeable exposure. Imaging methods count particles within instrument-specific size and spectral windows, Py-GC/MS quantifies polymer-specific mass in a processed sample fraction, and SEM-EDX provides morphology and elemental evidence without definitive molecular identification [8-18]. We therefore propose that every combination of analytical platform, size fraction, sample preparation, and reporting unit should be treated as a distinct exposure construct until biological reference materials and cross-platform calibration studies demonstrate equivalence. This platform-aware interpretation can explain differing polymer rankings without assuming that one study is necessarily biologically inconsistent with another, and it argues against pooling particle counts with polymer-mass concentrations [8-18].

Three Non-exclusive Biological Pathways

The reviewed evidence supports three non-exclusive pathways by which plastic-associated exposure could influence the ovarian follicle. The first is a direct particle pathway, in which sufficiently small particles interact with cumulus cells, granulosa cells, oocytes, or follicular tissue; engineered-particle imaging and experimental ovarian studies provide proof of biological plausibility under defined exposure conditions [15,19-30]. The second is a chemical-cargo or mixture pathway, in which additives, adsorbed contaminants, or co-exposures contribute to toxicity that cannot be attributed to the polymer backbone alone [4,23,34]. The third is an indirect systemic pathway mediated by intestinal-barrier injury, altered microbial metabolites, inflammation, vascular responses, or endocrine signaling, as illustrated by the mouse environment-gut-ovary model [14]. These pathways may operate simultaneously, so future human studies should measure particles together with selected additives, systemic inflammatory markers, and relevant microbial or metabolic signals rather than treating polymer detection as an isolated exposure [4,14,15,19-30,34].

The Follicular-Fluid Concentration Paradox

We propose a concentration-related interpretive hypothesis: a value measured at oocyte retrieval may reflect external exposure and systemic distribution, but it may also vary with follicle size and aspirated fluid volume, follicular protein or lipid composition, vascular permeability, ovarian stimulation, or local clearance. The dynamic composition of follicular fluid and the selective blood-follicle barrier make these influences biologically plausible, although their effects on measured microplastic concentrations have not been established [1,5,8,10,12-14]. This creates what we term a concentration paradox in diminished ovarian reserve: a higher measured polymer concentration could contribute to impaired follicular function, arise partly from altered follicular physiology, or reflect both processes [8,10,12-14]. The available case-control studies cannot distinguish these directions because exposure and ovarian-reserve status were assessed in the same treatment cycle [13,14]. To address this problem, future protocols should evaluate reporting polymer mass or particle number, alongside aspirated fluid volume, follicular dimensions, blood contamination, total protein or lipid content, paired systemic exposure markers, and repeated follicle-level samples. Such normalization strategies require validation and should be compared prospectively rather than assumed to represent cumulative dose [8,12-18].

A Staged Evidence-to-Clinical Translation Framework

On the basis of the reviewed studies, we propose a five-stage framework for evaluating progress toward clinical application. Stage 1 is analytical validity: recovery, blanks, detection limits, polymer specificity, and reproducibility must be demonstrated. Stage 2 is reproducible biological presence across laboratories and populations. Stage 3 is an adjusted association with a prespecified reproductive endpoint. Stage 4 requires prospective temporality, an interpretable dose-response pattern, and convergence with human biomarkers or mechanistic evidence at relevant internal doses. Stage 5 requires clinical utility, including a validated threshold and evidence that testing or an intervention improves decision-making or outcomes [8-18].

The current literature has achieved parts of stages 1-3 but not stage 4 or 5. Detection has been replicated with several platforms, and selected studies report associations with fertilization or diminished ovarian reserve; however, analytical equivalence, the independent replication of effect estimates, prospective temporality, and actionable thresholds remain absent [8-18]. This framework clarifies why biological plausibility should accelerate targeted research without prompting premature clinical testing, individualized risk prediction, or treatment recommendations.

Limitations of the Current Evidence

The direct human literature is young and heterogeneous. Most full studies were single-center, and several included fewer than 50 women. ART populations are selected for infertility and ovarian stimulation, limiting generalization to naturally conceiving women. Case-control recruitment can introduce selection bias, and cross-sectional follicular-fluid sampling cannot establish exposure timing. Age matching or multivariable adjustment reduces but does not eliminate confounding [6-14].

Exposure measurement remains a central limitation. Studies examined different size ranges and reported particles/mL, counts per analyzed area, mg/kg, or µg/g. Polymer profiles varied with instrument, spectral library, digestion, and filtration. Some methods supplied molecular identification but not size, whereas others supplied morphology but incomplete polymer confirmation. Blank strategies were inconsistent, and clinical collection materials remain difficult to control. These differences preclude quantitative pooling [8-18].

Outcome measurement was also variable. Reported endpoints included FSH, AMH, antral follicle count, oocyte yield, fertilization, cleavage, embryo quality, implantation, pregnancy, miscarriage, and live birth. Small samples and multiple comparisons increase false-positive and false-negative risks. Follicular-fluid concentrations may reflect follicle physiology or stimulation-related concentration changes rather than cumulative systemic dose. Reverse causation is especially plausible in DOR, where altered follicular number, vascularity, or fluid composition could affect the measured concentration [8,10,12-14].

Finally, in vivo animal and in vitro cell studies commonly use selected polymers and exposure conditions with uncertain relevance to human internal doses. Publication bias toward harmful results cannot be excluded. The reviewed literature supports hazard hypotheses and biological plausibility, but no human study has yet demonstrated a dose-response relationship replicated across independent laboratories with prospective temporality and clinically meaningful fertility endpoints [4,8-14,19-30].

Future directions

The next phase should move from isolated detection studies to coordinated exposure epidemiology. A consensus protocol should define follicular-fluid collection, first-versus-later follicle sampling, blood-contamination exclusion, storage, digestion, blank frequency, recovery, polymer reporting, and minimum metadata. Samples should be collected with paired blood, urine, and, where feasible, household dust or diet measures to clarify exposure sources and pharmacokinetics. Orthogonal imaging and mass methods should be applied to a shared subset, and blinded interlaboratory comparisons should establish reproducibility [16-18].

Prospective multicenter cohorts should enroll women before stimulation, record infertility diagnosis and treatment in detail, and preregister primary outcomes. Follicle-specific sampling can link exposure with oocyte maturity, fertilization, embryo development, aneuploidy where clinically tested, and cumulative live birth. Analyses should account for clustering within women, missing transfers, male-partner factors, and competing clinical decisions. Repeated cycles could help assess within-person stability and temporal ordering [8,10,12-14].

DOR research should use explicit diagnostic criteria and follow ovarian-reserve trajectories rather than relying only on case-control comparisons. Key confounders include age, body mass index, smoking, socioeconomic status, occupation, dietary patterns, drinking-water source, air pollution, plastic use, endocrine disorders, endometriosis, and co-exposure to plasticizers and persistent pollutants. Mixture models may be more appropriate than single-polymer regressions [13,14,31,32,34].

Mechanistic work should prioritize environmentally weathered particles, polymer mixtures observed in human follicular fluid, measured internal-dose ranges, and long-term low-dose exposure. Models should distinguish direct particle entry from indirect systemic or gut-mediated effects. Human primary granulosa and cumulus cells, follicle culture, organoids, and physiologically based toxicokinetic models could improve translation. Candidate mechanisms should be tested with intervention or rescue designs and validated in human biospecimens [4,14,19-30].

Finally, the field needs reference materials and reporting guidance specific to biological matrices. Data repositories containing spectra, particle images, pyrolysis markers, blank values, and patient-level de-identified outcomes would support reanalysis. Until these foundations are established, replication and method comparison are more valuable than additional small detection-only series [16-18].

## Conclusions

The available literature supports the presence of plastic-derived particles or polymer mass in human ovarian follicular fluid. Six full observational studies published by July 28, 2026, including a couple-based in vitro fertilization study and two case-control studies of diminished ovarian reserve, extend the evidence beyond initial proof-of-concept reports. Several studies observed associations with follicle-stimulating hormone, fertilization, anti-Müllerian hormone, antral follicle count, or diminished ovarian reserve, while others found no association with selected outcomes. Analytical heterogeneity, small samples, cross-sectional designs, and incomplete control of clinical and environmental confounding mean that these findings are not yet clinically predictive and do not establish causation.

Animal, follicle, oocyte, and granulosa-cell studies provide plausible pathways involving oxidative stress, inflammation, cell death, steroidogenesis, mitochondrial function, meiosis, and gut-ovary signaling. The integrative interpretation developed in this review treats current follicular-fluid burden as a measurement-defined construct and distinguishes direct particle effects, chemical-cargo effects, and indirect systemic pathways. A staged evidence-to-clinical framework places the field between replicated detection and preliminary association, before prospective causal inference or demonstrated clinical utility. The immediate priorities are standardized contamination control, orthogonal measurement, multicenter longitudinal cohorts, follicle-level reproductive outcomes, and independent replication. At present, follicular-fluid microplastic measurement should remain a research tool rather than a diagnostic test or basis for fertility treatment.
